# The rostromedial tegmental nucleus and alcohol addiction

**DOI:** 10.18632/oncotarget.15822

**Published:** 2017-03-01

**Authors:** Jiang-Hong Ye, Rao Fu, Wen He

**Affiliations:** Department of Anesthesiology, Rutgers, The State University of New Jersey, New Jersey Medical School, New Jersey, USA

**Keywords:** alcohol, addiction, mu opioid receptor

The neurobiological mechanisms underlying alcohol addiction remain obscure. It is generally accepted that alcohol’s addictive property is associated with its ability to increase the activity of dopaminergic (DA) neurons in the ventral tegmental area (VTA) in the brain. These neurons are under the powerful control of synaptic inputs. Thus, the synaptic regulation of DA neurons is a key initial step in reward mechanisms leading to alcohol addiction. DA neurons receive major GABAergic afferents. Some drugs of abuse, such as opioids, stimulate VTA-DA neurons through suppression of GABAergic transmission - that is by disinhibition. Emerging evidence indicates that the rostromedial tegmental nucleus (RMTg), a recently defined structure with dense Mu-opioid receptor (MOR) immunoreactivity, is a major GABAergic afferent to DA neurons, and a key structure in MOR-dependent regulation of DA neurons [1]. Substantial evidence associates the endogenous opioid system with the development and maintenance of alcoholism [2]. Alcohol alters the activity of endogenous opioid peptides, including biosynthesis, release, and degradation, as well as binding to opioid receptors, and these alterations may modulate many of the alcohol-related behaviors, including drinking and conditioned place preference (CPP). Blocking MORs with naltrexone, an opioid antagonist significantly decreases alcohol intake in preclinical settings and in humans with inconstant efficacy. However, the underlying mechanisms connecting MOR expressing RMTg neurons with alcohol drinking behavior remain obscure.

In an attempt to address this issue, in a recent study[3], we measured alcohol related behaviors after lesioning the RMTg with a special pharmacological agent, dermorphin-saporin (DS). Dermorphin is a potent MOR agonist, causing MOR internalization upon binding[4], whereas saporin is a ribosome-inactivating cytotoxin [5]. Microinjection of DS into the RMTg substantially reduced the number of RMTg cells. Importantly, the rats that received DS injection elevated their alcohol intake and preference compared to those that received an injection of blank saporin (BS), which did not cause neuronal damage. Based on these results, and the evidence from previous rodent studies suggesting that alcohol’s aversive effects limit voluntary intake, we propose that MOR-expressing GABAergic neurons in the RMTg may be an important structure of alcohol aversion. Conversely, given that alcohol’s euphoric effect encourages drinking, which may be normally constrained by these neurons; hence the ablation of these neurons makes drinking more rewarding. To test these hypotheses, we conduced a conditioned place preference (CPP) experiment, and found that rats spent more time in the chamber where they received intraperitoneal injections of alcohol (2g/kg), implying that alcohol gave them a rewarding feeling. Rats that received intra-RMTg DS treatment, compared to those that received BS treatment spent more time in the chamber associated with alcohol administration, suggesting that alcohol likely became more rewarding, and/or less aversive. The results from these two independent alcohol-related behavioral studies (voluntary drinking and CPP) suggest that alcohol is more reinforcing or less aversive to rats with RMTg destruction, and that MOR expressing neurons in the RMTg may influence the balance of rewarding/aversion effects of alcohol.

Our results are in line with a recent rat study showing that excitotoxic lesion of the RMTg using quinolinic acid increases voluntary ethanol consumption and accelerates extinction of ethanol-induced conditioned taste aversion [6]. Our results are also in line with previous rodent studies that associated the RMTg with aversion, including that driven by drug rewards [7, 8], and that inhibition of VTA-GABAergic neurons increases VTA-DA neuronal activity and is behaviorally rewarding. Conversely, direct excitation of VTA-GABA neurons disrupts reward-related behaviors, and stimulation of VTA-GABAergic neurons or inhibition of VTA-DA neurons promotes aversion [9].

We additionally found that intra-RMTg injection of DS increased locomotion of rats, implying that locomotion may normally be limited by the RMTg. Since it has been well documented that dopamine has a significant role in locomotion, and inhibition of RMTg neurons disinhibits VTA-DA neurons [1], we propose that the hyperactivity of rats may be resulted from the increased DA activity.

There are limitations of current study. For example, areas surrounding the RMTg having moderate levels of MOR expression could have been damaged by intra-RMTg DS injection. To minimize this risk, we injected DS of a small volume and a low dose. Despite these precautions, there were lesions outside the RMTg. Therefore, we cannot conclude that all the changes in alcohol-related behaviors induced by intra-RMTg DS injection were exclusively a result of RMTg cell damage. And while the current evidence suggests that increased dopamine may contribute to the hyperactivity of rats with RMTg ablation, direct evidence supporting this possibility is still lacking.

In summary, we demonstrated that damage of RMTg MOR-expressing GABAergic neurons by DS increased the intake and preference for alcohol, boosted the expression and slowed down the extinction of alcohol CPP, and increased locomotion. These were not seen in rats receiving BS or vehicle injection. These results indicate that the RMTg plays an important role in the regulation of voluntary alcohol drinking and CPP, as well as locomotion.
